# Antibiotic dose and nutrient availability differentially drive the evolution of antibiotic resistance and persistence

**DOI:** 10.1093/ismejo/wrae070

**Published:** 2024-05-01

**Authors:** Etthel M Windels, Lloyd Cool, Eline Persy, Janne Swinnen, Paul Matthay, Bram Van den Bergh, Tom Wenseleers, Jan Michiels

**Affiliations:** VIB Center for Microbiology, Flanders Institute for Biotechnology, Kasteelpark Arenberg 20, 3001 Leuven, Belgium; Centre of Microbial and Plant Genetics, KU Leuven, 3001 Leuven, Belgium; Department of Biosystems Science and Engineering, ETH Zürich, 4056 Basel, Switzerland; Swiss Institute of Bioinformatics, 1015 Lausanne, Switzerland; VIB Center for Microbiology, Flanders Institute for Biotechnology, Kasteelpark Arenberg 20, 3001 Leuven, Belgium; Centre of Microbial and Plant Genetics, KU Leuven, 3001 Leuven, Belgium; Laboratory of Socioecology and Social Evolution, KU Leuven, 3000 Leuven, Belgium; Centre of Microbial and Plant Genetics, KU Leuven, 3001 Leuven, Belgium; Centre of Microbial and Plant Genetics, KU Leuven, 3001 Leuven, Belgium; VIB Center for Microbiology, Flanders Institute for Biotechnology, Kasteelpark Arenberg 20, 3001 Leuven, Belgium; Centre of Microbial and Plant Genetics, KU Leuven, 3001 Leuven, Belgium; VIB Center for Microbiology, Flanders Institute for Biotechnology, Kasteelpark Arenberg 20, 3001 Leuven, Belgium; Centre of Microbial and Plant Genetics, KU Leuven, 3001 Leuven, Belgium; Laboratory of Socioecology and Social Evolution, KU Leuven, 3000 Leuven, Belgium; VIB Center for Microbiology, Flanders Institute for Biotechnology, Kasteelpark Arenberg 20, 3001 Leuven, Belgium; Centre of Microbial and Plant Genetics, KU Leuven, 3001 Leuven, Belgium

**Keywords:** antibiotics, resistance, persistence, experimental evolution, mathematical modeling

## Abstract

Effective treatment of bacterial infections proves increasingly challenging due to the emergence of bacterial variants that endure antibiotic exposure. Antibiotic resistance and persistence have been identified as two major bacterial survival mechanisms, and several studies have shown a rapid and strong selection of resistance or persistence mutants under repeated drug treatment. Yet, little is known about the impact of the environmental conditions on resistance and persistence evolution and the potential interplay between both phenotypes. Based on the distinct growth and survival characteristics of resistance and persistence mutants, we hypothesized that the antibiotic dose and availability of nutrients during treatment might play a key role in the evolutionary adaptation to antibiotic stress. To test this hypothesis, we combined high-throughput experimental evolution with a mathematical model of bacterial evolution under intermittent antibiotic exposure. We show that high nutrient levels during antibiotic treatment promote selection of high-level resistance, but that resistance mainly emerges independently of persistence when the antibiotic concentration is sufficiently low. At higher doses, resistance evolution is facilitated by the preceding or concurrent selection of persistence mutants, which ensures survival of populations in harsh conditions. Collectively, our experimental data and mathematical model elucidate the evolutionary routes toward increased bacterial survival under different antibiotic treatment schedules, which is key to designing effective antibiotic therapies.

## Introduction

Under repeated exposure to antibiotic stress, many bacterial strains rapidly acquire genetic mutations that render them resilient to treatment. This evolutionary adaptation has become a popular subject of study, offering fundamental insights into bacterial evolution as well as guiding the response to the burning antibiotic resistance crisis [1].

Two major evolutionary strategies have been observed in bacterial populations adapting to antibiotic treatment: resistance and tolerance. Resistance refers to the ability of bacteria to grow at elevated drug concentrations, thus preventing effective clearance of infections [2]. Resistance is conferred by genetic alterations that result in degradation or inactivation of the antibiotic, antibiotic efflux, or drug target modification [3] and can be quantified using the minimum concentration of an antibiotic required to inhibit growth of a bacterial strain (minimum inhibitory concentration; MIC). In contrast, antibiotic tolerance allows bacteria to temporarily endure or slow down the lethal consequences of bactericidal antibiotics, presumably without growing in their presence [2]. Tolerance implies both population-wide tolerance, where the bacterial population consists exclusively of antibiotic-tolerant cells, and persistence, where only a subpopulation of cells shows increased tolerance. In the case of persistence, the antibiotic-tolerant cells in the population are also denoted as persister cells. These persister cells are genetically identical to, but phenotypically different from the sensitive cells in the population. Persistence can be quantified using the persistence level, corresponding to the fraction of persister cells in the population. Increased tolerance can be induced by stressful environmental conditions, but can also result from genetic mutations [4, 5].

Tolerant cells are assumed to be nondividing in the presence of antibiotics, whereas resistant mutants can grow in antibiotic concentrations exceeding the MIC of the susceptible strain [2]. Based on this important distinction, we anticipated that the availability of nutrients during antibiotic treatment would favor resistance over persistence, because the latter is associated with a growth deficit [6]. Additionally, growth inhibition of resistant mutants by antibiotics is highly dose-dependent, as these mutants can cope with antibiotic doses below a specific threshold (i.e. their MIC) but not above. Single resistance mutations often elevate this threshold only slightly above the MIC of the susceptible strain. In contrast, tolerant cells are able to endure a wide range of drug concentrations. Hence, repeated treatment with high antibiotic concentrations is expected to favor tolerance over resistance, as these concentrations might be difficult to overcome by a single resistance-conferring mutation. Intermitting these treatments with antibiotic-free phases is crucial to allow antibiotic-tolerant cells to resume growth. Several studies accordingly revealed strong selection for tolerance when populations are intermittently exposed to lethal antibiotic doses [7–13], whereas continuous exposure to sublethal antibiotic doses in nutrient-rich conditions has been shown to select for resistance [14–16]. Despite these efforts, a comprehensive view on the role of nutrient and antibiotic concentrations in resistance and tolerance evolution is currently lacking.

Resistance and tolerance evolution are often studied in isolation, and only a handful of studies have brought both into the same picture. Among those, one early study revealed a positive correlation between resistance and persistence levels in environmental *Pseudomonas* isolates, suggesting some degree of complementarity between both strategies [17]. Correspondingly, resistance and tolerance mutations were later shown to act synergistically on antibiotic survival [18]. Another line of research demonstrated that increased tolerance can promote resistance evolution, as tolerant cells remain viable during antibiotic treatment and might serve as a reservoir from which resistant mutants can emerge [19–23]. However, it has remained unclear in which specific treatment conditions such interplay drives evolutionary adaptation.

In this study, we adopted a spectrum of antibiotic treatment profiles expected to promote different degrees of resistance and persistence evolution. Taking the distinct growth and dose–response characteristics of resistance and persistence mutants as a premise, we investigated the effects of varying antibiotic doses and nutrient levels during treatment on the evolutionary route toward high antibiotic survival. We aimed to identify which survival strategy thrives under each treatment regimen and to investigate how the strategies relate. To this end, we used a high-throughput protocol to monitor hundreds of experimentally evolving lines of *Escherichia coli* intermittently exposed to antibiotics, simultaneously probing a broad range of selection conditions. These experimental conditions were mimicked with a mathematical model to further explore the determinants of evolutionary adaptation.

Our results demonstrate that resistance evolution is more prominent at high nutrient levels, whereas persistence is favored under low-nutrient conditions. Resistance evolution in itself is mostly impeded at high antibiotic concentrations, but preceding or concurrent evolution of persistence can sustain partially resistant mutants in such high-dose treatment conditions. Our simulations suggest that these observations can be largely explained by the different growth response of resistant and persister cells to antibiotics and nutrients.

## Materials and methods

### Strains and culture conditions

All experiments were initiated from the *E. coli* SX43 strain. This strain has the BW25993 background, but additionally expresses a Tsr-Venus fusion from the *lacZ* locus, resulting in yellow fluorescence at the cell poles [24]. Cultures were grown at 37°C in Mueller–Hinton broth (MHB) with orbital shaking (1.5-mm shaking orbit; 1200 rpm; Titramax 1000, Heidolph Instruments, Schwabach, Germany) or on lysogeny broth (LB) agar.

### Evolution experiments

Experimental evolution was initiated by inoculating single colonies of the ancestor strain. Per treatment condition, 24 parallel populations were initiated and evolved for 11–16 days. Populations were grown in 96-well plates filled with 500 μl MHB and treated daily for 5 h with varying concentrations of amikacin, after transferring 200 μl of culture to a fresh 96-well plate. Before treatment initiation, the stationary phase cultures were diluted in fresh MHB at a culture-to-total volume ratio of 3:4, 1:2, or 1:5, resulting in a nutrient level of 0.25, 0.5, or 0.8, respectively. Following treatment, the cultures were washed three times in 10-mM MgSO_4_ to remove the antibiotic and subsequently diluted 1:100 in fresh MHB. Before and after each treatment, 10-fold dilution series were prepared in MgSO_4_ and the cell density of the cultures was determined by spot plating on LB agar. Plates were incubated for 24 h before colonies were counted.

### Measurement of minimum inhibitory concentrations

MIC values were measured using the microdilution method [25]. Cultures were initiated from single colonies, grown overnight, and subsequently diluted in MHB to obtain a cell density of 10^5^ CFU/ml. These inocula were incubated in a range of twofold amikacin dilutions. After 24 h, growth was examined visually through optical density measurement at 595 nm (Synergy Mx Microplate Reader, BioTek, Santa Clara, US). The MIC value was defined as the lowest antibiotic concentration in which no growth was detected. Four technical replicates were tested per population.

### Measurement of persistence levels and time–kill curves

An overnight culture initiated from a single colony was diluted 1:100 in 500 μl MHB and again grown overnight for 16 h. Unless stated otherwise, this culture was diluted 3:4 in fresh MHB and treated with 100 μg/ml amikacin or, for highly resistant strains (MIC ≥ 16 μg/ml), 400 μg/ml amikacin. After treatment, cultures were washed in 10 mM MgSO_4_ to remove the antibiotic. For persistence assays, cell densities (CFU/ml) were determined before treatment and after 5 h of treatment, by preparing 10-fold dilution series in MgSO_4_ followed by plating on LB agar. Persistence levels were defined as the ratio of the cell density after and before 5 h of treatment. For time–kill curves, cell densities were measured before treatment and after 1, 2, 3, 5, and 8 h of treatment. Plates containing untreated cultures were incubated for 24 h, whereas plates containing treated cultures were incubated for 48 h before colonies were counted. Three biological replicates were tested per population.

### Model description

Evolutionary dynamics of well-mixed bacterial populations were modeled stochastically using a discrete-time algorithm written in MATLAB (MathWorks). Spatial effects on cell behavior and physical interactions between cells are not considered in the model. Cells can belong to 30 different mutant classes, with each class being characterized by a combination of a log_2_(MIC) from the set {1,2,3,4,5,6} and a log_10_(switching rate to persister state) from the set {-4.5,-3.5,-2.5,-1.5,-0.5}, with the lowest values of these sets corresponding to the ancestral levels. Cells in each class can reproduce, die, switch to and from the persister phenotype, and mutate, with class-specific rates. Antibiotic and nutrient concentrations vary through time, in accordance with our experimental setup. All model parameter values are based on experimental observations and literature, and are summarized in Table S1.

#### Population dynamics

The rate of change in cell count for regular cells belonging to a specific mutant class is determined by the class-specific net growth rate, switching rates to and from the persister state, and mutation rates to and from other classes. We assumed that persister cells do not grow, are not killed by antibiotics, and do not mutate. Population dynamics are simulated using a binomial distribution-based τ-leap approximation of the Gillespie algorithm [26], with a 30-min time interval. Initially, all mutant classes are empty except for the ancestral class, which contains 10^3^ regular cells and 1 persister cell. The carrying capacity is set to 10^6^ cells for computational feasibility.

#### Growth and killing

Growth and killing are described with a net growth rate, because bulk experiments do not provide estimates for individual replication and death rates. As derived in [27], the dependence of the net growth rate of a bacterial population (described by the change in log_10_[cell count] per hour) on the antibiotic concentration can be described with a four-parameter pharmacodynamic function, based on the Hill function:


$$ {\mathrm{\psi}}_i(AB)={\mathrm{\psi}}_{max}-\frac{\psi_{max}-{\mathrm{\psi}}_{min}}{{\left(\frac{AB}{MIC_i}\right)}^{\kappa_{AB}}-\left(\frac{\psi_{min}}{\psi_{max}}\right)}{\left(\frac{AB}{MIC_i}\right)}^{\kappa_{AB}} $$

where $AB$ represents the antibiotic concentration, ${\mathrm{\psi}}_{max}$ and ${\mathrm{\psi}}_{min}$the maximum and minimum growth rate, and ${MIC}_i$ the MIC associated with mutant class $i$. The Hill coefficient ${\kappa}_{AB}$ describes the sensitivity of the bacterial growth rate to changes in the antibiotic concentration. The equation above implies that ${\mathrm{\psi}}_i(AB)$ is positive (growth) when $AB<{MIC}_i$ and negative (killing) when $AB>{MIC}_i$.

Because ${\mathrm{\psi}}_i(AB)$ is defined as the change in log_10_(cell count) per hour, it represents a net population growth rate [27]. In other words, the change in the log_10_(cell count) occurring during $\Delta t$ equals


$$ {\mathit{\log}}_{10}\left({N}_2\right)-{\mathit{\log}}_{10}\left({N}_1\right)={\mathit{\log}}_{10}\left(\frac{N_2}{N_1}\right)={\mathrm{\psi}}_i(AB)\Delta t $$

with ${N}_1$ and ${N}_2$ the population sizes at the start and end of $\Delta t$, respectively. During the growth phase, which corresponds to cell doubling, the number of divisions per cell occurring during $\Delta t$ equals


$$ {\mathit{\log}}_2\left(\frac{N_2}{N_1}\right)={\mathit{\log}}_2\left({10}^{\psi_i(AB)\Delta t}\right) $$

For small $\Delta t$, this equals the per-cell probability to divide within $\Delta t$. This probability is corrected to account for nutrient dependence:


$$ {g}_i\left( AB,n\right)={\mathit{\log}}_2\left({10}^{\psi_i(AB)\Delta t}\right)n \ \text{for}\ {\psi}_i(AB)>0. $$

with $n$ the nutrient level (representing the nutrient concentration relative to the concentration in 100% fresh medium, thus varying between 0 and 1), which is used as a logistic growth factor (because $n=\frac{K-N}{K}$, with $K$ the carrying capacity and $N$ the population size).

Similarly, for the antibiotic killing phase, the probability of death per cell in $\Delta t$ is calculated assuming an exponential population size decay:


$$ {g}_i\left( AB,n\right)=1-{10}^{\psi_i(AB)\eta (n)\Delta t} \ \text{for}\ {\mathrm{\psi}}_i(AB)<0 $$

with $\eta (n)=\frac{1}{1+{\left(\frac{0.5}{n}\right)}^{\kappa_n}}$ describing the dependence of the killing rate on the nutrient concentration (corresponding to our experimental time–kill data; Fig. S1). Similar to the dependence of the killing rate on the antibiotic concentration, this nutrient dependence is modeled as a Hill function.

We set ${\mathrm{\psi}}_{max}=0.6\ {h}^{-1}$, which corresponds to an average of one division per cell per 30 min (at $AB=0$ μg/ml and $n=1)$ for the ancestor, and ${\mathrm{\psi}}_{min}=-10\ {h}^{-1}$, which corresponds to a change in log_10_(cell density) of approximately $-6\ {h}^{-1}$ (at $AB=400$ μg/ml and $n=1$). This is in accordance with our experimentally measured killing rate of non-persister cells, determined as the first-phase rate of a biphasic fit to time–kill data at high antibiotic and high nutrient concentrations (Fig. S1). ${\kappa}_{AB}$ and ${\kappa}_n$ were both set to 1 [27]. Together, this mathematical description and choice of parameter values result in modeled killing rates that match the experimental data relatively well (Fig. S2).

#### Persister formation

In accordance with other models of persistence, transitions between regular and persister cell states are modeled using forward and backward switching rates ${a}_i$ and $b$ [9, 28–31]. Stressful environmental conditions, including nutrient limitation, have been shown to increase rates of persister formation [32]. To simulate this increased switching to the persister state during late-exponential and stationary phase, the forward switching rate ${a}_i$ is assumed to linearly depend on the nutrient level:


$$ {a}_i={a}_{i,\mathit{\max}}\left(1-n\right) $$

with ${a}_{i,\mathit{\max}}$ the maximum switching rate, occurring in stationary phase [9, 30], for cells in mutant class $i$. In accordance with the second-phase killing rate observed in our experimentally measured time–kill curves (Fig. S1), the switching rate from the persister state to the regular state, $b$, also changes linearly with the nutrient level:


$$ b={b}_{max}n $$

with ${b}_{max}$ the maximum switching rate, occurring in early exponential phase. $b$ and ${b}_{max}$ are not class-specific, and ${b}_{max}$ was set equal to $0.1\ {h}^{-1}$ per cell, based on literature [9, 33] and our experimental data (Fig. S1).

#### Mutation

**Figure 1:**
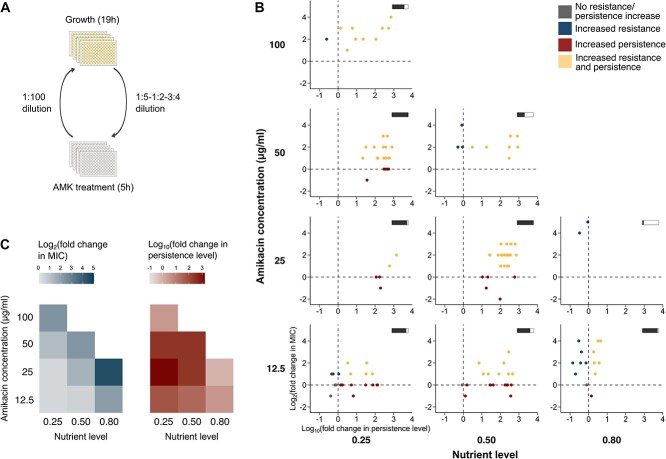
Resistance and persistence levels of experimentally evolved populations. (A) Setup of the evolution experiments. Populations founded from an ancestral *E. coli* strain (MIC = 2 μg/ml) were exposed to daily, 5-h treatments with varying amikacin (AMK) doses and nutrient levels, intermitted with periods of antibiotic-free growth. Nutrient levels were varied by diluting stationary phase cultures in fresh, nutrient-rich growth medium, using different dilution factors. In each condition, 24 parallel populations were evolved for 11–16 treatment cycles. (B) Resistance and persistence levels (expressed as log_2_- and log_10_-transformed fold changes relative to the ancestral level, respectively) of evolved populations, measured under the same conditions for all populations (see Materials and Methods). Horizontal, filled bars depict the proportion of populations that did not go extinct during the evolution experiment. Only populations for which resistance and persistence level measurements were available are shown. (C) Resistance and persistence levels averaged over all surviving populations per condition.

We only consider mutations that occur during DNA replication, implying that only newly generated daughter cells are allowed to mutate and stationary phase mutations, although potentially relevant, are ignored. Only beneficial mutations (i.e. mutations causing an increase in resistance or persistence) are considered, as deleterious mutations are assumed to rapidly get outcompeted. Although the model cannot describe systems where deleterious mutations are maintained through stochastic effects of genetic hitchhiking [34], this assumption is reasonable for our system under study, as strong decreases in persistence and resistance levels were not observed experimentally (Fig. 1B).

Cells in class $i$ that acquire a mutation are assigned to a different class $j$, with rate ${\mu}_{ij}$ defined as


$$ {\mu}_{ij}={10}^{\left({l}_i^R-{l}_j^R+{l}_i^P-{l}_j^P\right)} \ {for}\ i\ne j $$

where ${l}_i^R$ and ${l}_i^P$ correspond to the log_2_-transformed resistance level resp. log_10_-transformed persistence level, associated to class $i$. The equation above reflects an exponential distribution of fitness effects of beneficial mutations [35]. As deleterious mutations (i.e. mutations causing a decrease in resistance or persistence) are not considered, mutations only occur with ${l}_j^R\ge{l}_i^R$ and ${l}_j^P\ge{l}_i^P$. All ${\mu}_{ij}$ are scaled to obtain an overall rate of resistance and persistence mutations $m$. $m$ was set equal to 10^−4^ mutations per cell division, which is based on the mutation rate observed in experimental populations [13, 21], scaled to reflect a smaller population size and guarantee equal mutational supply.

#### Treatment regime

Mimicking the evolution experiments, antibiotic treatment in our simulations is initiated every 24 h and lasts 5 h, and is repeated for 10 evolutionary cycles. At the initiation of treatment, nutrients are added to the culture at a level ${n}_0$. ${n}_0$ represents the fraction of fresh medium added to a nutrient-exhausted culture and should thus be interpreted as the nutrient level relative to the maximal level in 100% fresh medium. After treatment, the population is transferred to fresh, nutrient-rich medium by setting the nutrient level equal to 1. During growth, the nutrient level declines proportional to the increase in population size:


$$ n\left({t}_2\right)-n\left({t}_1\right)=-\frac{N_{tot}\left({t}_2\right)-{N}_{tot}\left({t}_1\right)}{K} $$

with $n(t)$ denoting the nutrient level at time $t$ and ${N}_{tot}(t)$ the total bacterial population size at time $t$. It is worth noting that the growth yield coefficient (i.e. biomass-to-substrate yield) does not matter in this conversion, as the nutrient level is defined as a relative level that is 0 at maximal population size and 1 at low population size.

Before treatment, the population is diluted in fresh medium, with the dilution factor depending on the intended nutrient level. After treatment, populations are diluted 1:100 in fresh medium. In both cases, the population size is adjusted by drawing a sample from a multinomial distribution, with probabilities corresponding to the frequency of regular cells, persister cells, and dead cells in the different classes at the current time step.

### Data analysis

#### Data transformations

MIC values and persistence levels of populations are represented as fold changes relative to the ancestral level. These fold changes were log_2_-transformed in the case of MIC values and log_10_-transformed in the case of persistence levels. Survival levels were log_10_-transformed.

#### Statistical tests

The transformed variables were used as dependent variables in multivariable linear regression models with antibiotic and nutrient concentrations as continuous covariates.

The following linear model was fitted on the time–kill data:


\begin{align*} {\log}_{10}\left( survival\ after\ 8h\right)=\ {\beta}_0+{\beta}_1\left[ AMK\right]+{\beta}_2\left[ nutrient\right]\nonumber +{\beta}_3\left[ AMK\right]\left[ nutrient\right] \end{align*}

with $survival\ after\ 8h$ the ratio of the cell density after and before 8 h of treatment, and $\left[ AMK\right]$ and $\left[ nutrient\right]$ the amikacin and nutrient concentration, respectively.

For the experimental evolution data, the following models where fitted:

1) $ {\log}_{10}\left( surviving\ fraction\right)={\beta}_0+{\beta}_1\left[ AMK\right]+{\beta}_2\left[ nutrient\right] $
2) $ proportion\ of\ extinct\ populations={\beta}_0+{\beta}_1\left[ AMK\right]+{\beta}_2\left[ nutrient\right] $
3) $ {\log}_2(MIC)={\beta}_0+{\beta}_1\left[ AMK\right]+{\beta}_2\left[ nutrient\right] $
4) $ {\log}_{10}\left( persistence\ level\right)={\beta}_0+{\beta}_1\left[ AMK\right]+{\beta}_2\left[ nutrient\right] $

with $surviving\ fraction$ (the fraction of cells surviving the 5-h treatment), $proportion\ of\ extinct\ populations$, $persistence\ level$, and $MIC$ all defined at the end of the evolution experiment. Summary tables are provided in Tables S2–S6.

#### Time–kill curves

Biphasic killing parameters were determined by fitting a nonlinear mixed model to the log_10_-transformed, normally distributed fraction of surviving cells using the R package nlme (https://cran.r-project.org/web/packages/nlme/index.html). The model was based on the equation


$$ {\log}_{10}\left( surviving\ fraction\right)={\log}_{10}\left(\left(1-{P}_0\right){e}^{-{k}_n\tau }+{P}_0{e}^{-{k}_p\tau}\right) $$

with τ the treatment time (in hours), ${{P}}_{{0}}$ the initial fraction of persister cells (at τ = 0), and ${{k}}_{{n}}$ and ${{k}}_{{p}}$ the rate at which regular cells and persister cells are killed (per hour) [36]. Treatment condition was coded as a fixed factor and replicate was coded as a random factor.

## Results

### Selection of resistance and persistence is governed by the nutrient and antibiotic concentration

To experimentally study the combined effects of nutrients and antibiotic doses on the evolutionary route toward increased antibiotic survival, we evolved *E. coli* populations under daily, 5-h treatments with varying amikacin and nutrient levels, intermitted with periods of growth in antibiotic-free, nutrient-rich medium (see Materials and Methods; Fig. 1A). The nutrient level is defined here as the fraction of fresh medium in the culture (see Materials and Methods). The fraction of cells surviving the daily treatment was monitored over time (Fig. S3). The amikacin concentration and nutrient level during treatment were both positively correlated with the proportion of extinct populations (multivariable linear regression: *P* < .01 for both main effects; Table S4), which is in accordance with their observed effects on the killing efficacy (Fig. S1). With the exception of the condition with the lowest selective pressure, populations that did not go extinct showed strong evolutionary adaptation, as demonstrated by the drastic increase in antibiotic survival over time (Fig. S3).

**Figure 2:**
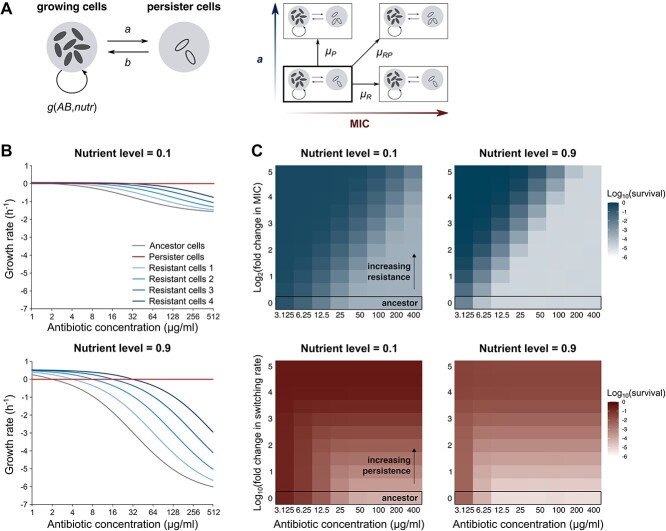
Modeled effects of nutrients and antibiotic doses on bacterial survival. (A) Schematic representation of the mathematical model of bacterial evolution. Cells can grow or die, with the net growth rate *g* depending on the antibiotic dose and nutrient level, and switch to and from the nongrowing persister state with rates *a* and *b*, respectively. Initially, all cells belong to the ancestral class, but they can mutate to a class with a higher persistence level (i.e. switching rate *a*) or resistance level (i.e. MIC value), or both, with mutation rates *μ_P_, μ_R_*, and *μ_RP_,* respectively. (B) Theoretical pharmacodynamic curves for ancestral cells (i.e. *E. coli* wild-type strain with MIC = 2 μg/ml) and different resistant cells with gradually increasing MIC (4–8-16–32 μg/ml). Growth rates represent the change in log_10_(cell density) per hour, with negative rates corresponding to cell death. The MIC of a strain corresponds to the antibiotic concentration for which the growth rate equals 0. Persister cells are assumed to neither grow nor die. (C) Simulated antibiotic survival of mutant populations after one treatment cycle. Simulations were initiated with isogenic populations of mutant strains with increasing resistance (*y*-axis in top graphs) or persistence (*y*-axis in bottom graphs) levels. These populations were exposed *in silico* to one cycle of growth (19 h) followed by antibiotic killing (5 h) with varying antibiotic concentrations at either low (left) or high (right) nutrient levels. *De novo* mutations were not considered in these simulations. MIC values and switching rates are expressed as log_2_- resp. log_10_-transformed fold changes relative to the ancestral level.

Resistance and persistence levels of evolved populations were determined at the end of the experiment (Fig. 1B and C). Many populations showed an increase in both resistance and persistence levels. The nutrient level was found to be positively correlated with the resistance level of evolved populations (multivariable linear regression: *P* < .001; Table S5), and populations showing only increased resistance were mainly observed at high nutrient levels in combination with low to intermediate antibiotic concentrations. In contrast, the nutrient level was negatively correlated with the persistence level of evolved populations (*P* < .001; Table S6), and populations showing only increased persistence were mainly observed at low to intermediate nutrient levels (Fig. 1B). Together, these results suggest that resistance mutants benefit from high nutrient levels and are favored over persistence mutants in these conditions. We did not identify a significant effect of the amikacin concentration on the persistence level of evolved populations (Table S6). However, it was positively correlated with the level of resistance (*P* < .001; Table S5) (Fig. 1C), which can be explained by an increased selective pressure at high amikacin concentrations. Nevertheless, observing resistance evolution at these high doses was unexpected, as single-step, low-level resistance mutants are only partially resistant to the applied dose and therefore, in theory, expected to be killed during antibiotic treatment (Fig. 2B). However, many of these evolved populations also exhibit increased persistence levels, which could explain why they survived the high-dose antibiotic treatments relatively well (Fig. S3). Partial resistance indeed requires to be complemented with persistence to ensure antibiotic survival and avoid population extinction. In contrast, persistence did not emerge as much in conditions where full resistance to the treatment can be easily obtained, i.e. at low antibiotic doses and high nutrient levels (Fig. 1B).

### Mathematical model capturing growth and survival dynamics of resistance and persistence mutants

We hypothesized that the observed effects of antibiotics and nutrients on resistance and persistence evolution can be explained by the different growth and antibiotic survival characteristics of resistance and persistence mutants, resulting in a differential fitness of such mutants. To test this hypothesis, we developed a mathematical model to simulate evolution of bacterial populations. Cells within each population can belong to different mutant classes, corresponding to different resistance and persistence levels (Fig. 2A). Within each class, cells replicate (positive growth rate) at sub-MIC antibiotic concentrations and get killed (negative growth rate) at supra-MIC concentrations, with the net growth rate depending on the antibiotic and nutrient concentration according to previously described Hill functions [27, 37] (see Materials and Methods). Furthermore, cells can switch from and to the antibiotic-tolerant persister state, which is associated with a zero growth rate in all conditions, and mutate to different classes. Population dynamics were simulated stochastically. The model parameters were either measured directly or taken from literature [9, 13, 21, 27, 33] (Table S1). Exceptions are the parameters relating the net growth rate to the antibiotic and nutrient concentration, which were informed by independently measured time–kill curves of the ancestral strain exposed to varying amikacin doses and nutrient levels (Figs S1 and S2).

The model input includes a mathematical description of our experimental observations regarding growth and antibiotic killing, and can be visually summarized as pharmacodynamic curves, demonstrating how the net growth rates of ancestral, resistant, and persister cells compare (Fig. 2B). To confirm that the description of these processes matches the experimental observations, we simulated survival of mutants that have gradually increasing resistance and persistence levels, exposed to a range of nutrient and antibiotic concentrations (Fig. 2C). As expected, these simulations appropriately capture how the survival of a resistance mutant is determined by the applied antibiotic concentration relative to the MIC of the mutant, while the effect of the antibiotic concentration on the survival of persistence mutants is limited. Furthermore, the nutrient-dependent killing, as observed experimentally (Fig. S1), seems well described by the model. Together, these results show that our model properly captures the characteristics of resistance and persistence regarding growth and antibiotic killing and can facilitate a detailed study of evolution under antibiotic stress.

**Figure 3:**
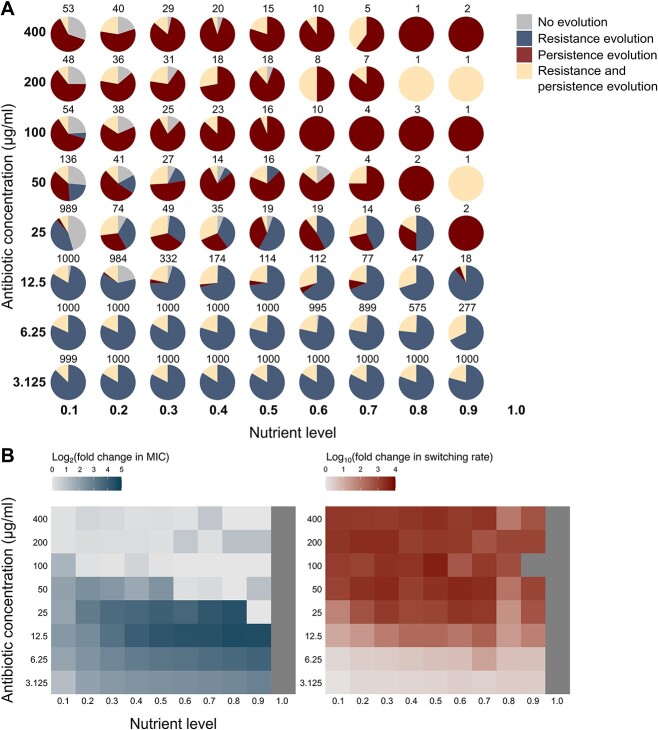
Resistance and persistence evolution in simulated populations. (A) Number of simulated populations evolving only resistance, only persistence, or both resistance and persistence, in various treatment conditions. 1000 populations were simulated per condition. The total number of populations that did not go extinct is indicated. (B) Resistance and persistence levels of evolved populations (expressed as log_2_- resp. log_10_-transformed fold changes relative to the ancestral level), averaged over all surviving populations per condition. Dark-gray squares correspond to conditions where all populations went extinct.

We used our model to investigate whether the distinct growth and survival dynamics of resistance and persistence mutants under antibiotic stress are sufficient to explain the experimentally observed evolutionary patterns. The model further allowed us to expand the range of treatment conditions and investigate evolutionary trajectories in more detail. Mimicking the experimental setup, we simulated evolution of bacterial populations intermittently exposed to varying antibiotic and nutrient concentrations (see Materials and Methods). Simulations under conditions characterized by a high nutrient and high antibiotic concentration resulted in a high proportion of extinct populations (Fig. 3A). Persistence was mainly selected at high antibiotic concentrations, whereas resistance was mainly selected at low antibiotic concentrations, with resistance levels increasing with increasing nutrient levels (Fig. 3B). High antibiotic doses only selected for resistance when accompanied with increased persistence (Fig. 3A). These patterns observed in the simulation output largely correspond to the experimental data, showing that our low-level model assumptions regarding growth and survival of resistance and persistence mutants are sufficient to reproduce the predominant high-level evolutionary trends.

### Resistance and persistence interplay at high antibiotic doses

**Figure 4:**
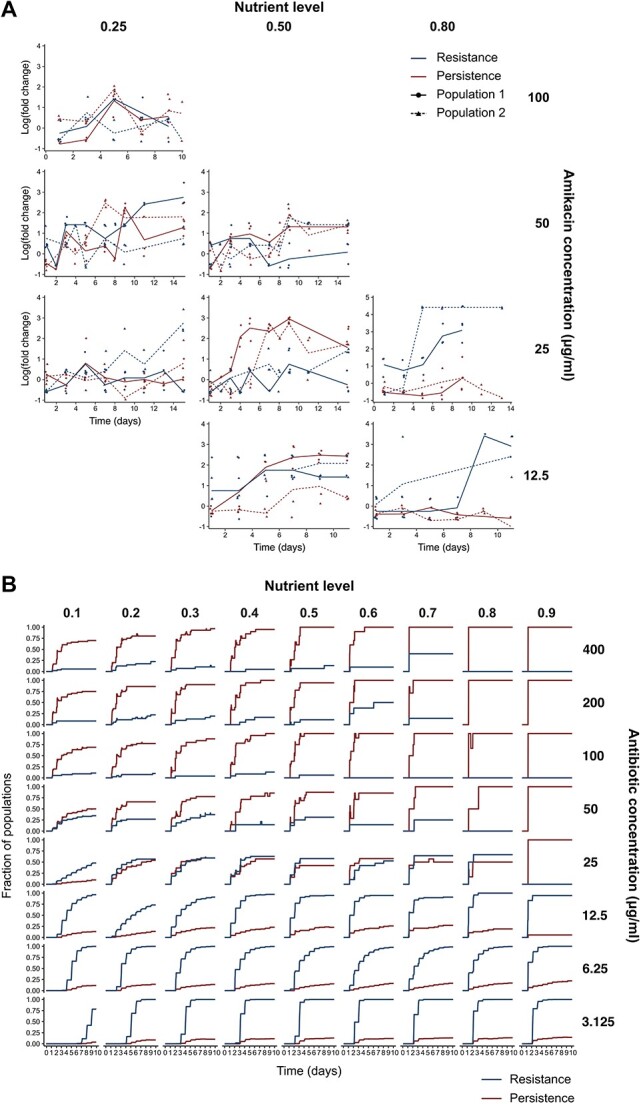
Resistance and persistence over time in experimentally evolved and simulated populations. (A) Resistance and persistence levels over time of experimentally evolved populations. Phenotypes were assessed at different time points by measuring persistence and resistance levels of three clones for two populations per condition. No measurements were performed for populations evolved with 12.5 μg/ml amikacin and a nutrient level of 0.25, as no evolutionary adaptation was observed for this condition (Fig. S3). Log(fold change) corresponds to log_10_(fold change) for persistence levels and log_2_(fold change) for resistance levels. Solid lines connect the means on log scale through time. (B) Fraction of simulated populations with increased resistance or persistence. A population was classified as having evolved resistance or persistence when at least 30% of the cells resided in mutant classes characterized by increased resistance or persistence, respectively. Only populations that survived until the end of the simulation are shown. The resistance and persistence curves overlap for the following conditions: 50 μg/ml—0.9; 200 μg/ml—0.8; 200 μg/ml—0.9.

**Figure 5:**
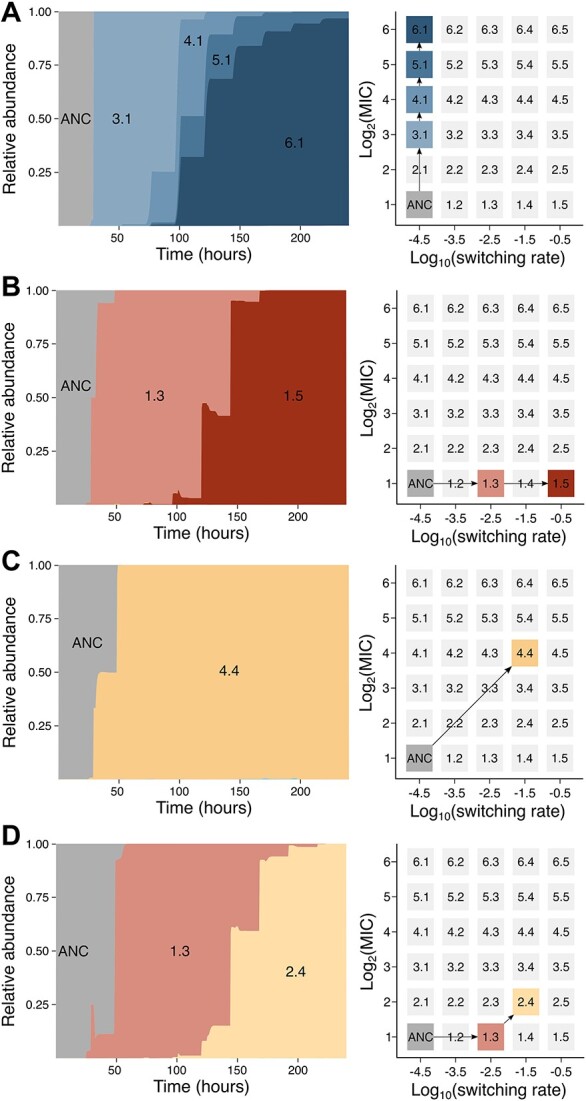
Evolutionary trajectories for simulated populations. Muller plots and corresponding evolutionary paths for a set of simulated populations, representative for different scenarios: (A) only resistance evolved (antibiotic concentration = 12.5 μg/ml; nutrient level = 0.8), (B) only persistence evolved (antibiotic concentration = 400 μg/ml; nutrient level = 0.6), (C) persistence and resistance evolved simultaneously (antibiotic concentration = 100 μg/ml; nutrient level = 0.4), and (D) persistence evolved first, followed by an increase in resistance and persistence (antibiotic concentration = 200 μg/ml; nutrient level = 0.6).

We further explored how resistance and persistence interact within experimentally evolved as well as simulated populations. Measurements of resistance and persistence levels of clones in the experimentally evolved populations at several intermediate time points are noisy, but suggest that an increase in resistance is most often accompanied with an increase in persistence (Fig. 4A). Exceptions are conditions characterized by high nutrient levels (0.80) and relatively low antibiotic doses (12.5–25 μg/ml), where resistance is selected independently of persistence. An initial rise in resistance and persistence can be followed by a decrease in persistence (Fig. 4A, upper graph). Similar behavior has been reported before [23] and could explain the low persistence levels observed in some end populations evolved at high antibiotic doses (Fig. 1B). The evolutionary trajectories for simulated populations, summarized per condition, further indicate that resistance easily evolves at low antibiotic doses, with the rate of resistance evolution increasing with increasing nutrient level (Fig. 4B). At high antibiotic doses, resistance evolution only occurs when accompanied with persistence evolution. One of the key advantages of our modeling approach is that complete information is provided on the mutational dynamics in all individual populations over time. The detailed evolutionary trajectories of a few selected populations, representative for different scenarios, illustrate this point (Fig. 5). These trajectories suggest that resistance evolution at high antibiotic doses either occurs through the emergence of a single mutation with pleiotropic effects on resistance and persistence or through the emergence of a persistence mutation followed by a resistance or pleiotropic mutation (Fig. 5C and D).

To establish the critical role of persistence in the survival and evolution of populations subjected to high antibiotic doses, we simulated resistance evolution without allowing for evolution of persistence. This implies that mutations were assumed to only affect resistance levels, while persistence was maintained at the ancestral level. A drastically higher proportion of extinct populations was observed in this case: at antibiotic concentrations above 25 μg/ml and nutrient levels above 0.3, the absence of persistence evolution results in a drop in the total number of surviving populations from 207 to 3 (Fig. 3A; Fig. S4). Selection for resistance only occurred at low to intermediate antibiotic concentrations, as only in these conditions, first-step resistant mutants have a chance of surviving and being selected (Fig. S4). Overall, these simulations indicate that persistence evolution ensures survival of populations at high antibiotic doses, facilitating resistance evolution in these conditions.

## Discussion

Although resistance and tolerance have both been identified as important causes of antibiotic treatment failure [38, 39], there is limited knowledge as to which selective pressures drive their evolution. The goal of this study was to elucidate how different antibiotic treatment conditions govern the selection of resistance and persistence. As resistant and persister cells mainly differ in their growth characteristics and in their response to increasing drug concentrations, we focused on the effects of nutrient levels and antibiotic doses on the evolutionary routes toward high antibiotic survival. We investigated these effects using large-scale experimental evolution and built a mathematical model to gain insight into the factors underlying the observed evolutionary patterns.

As resistant mutants can grow during antibiotic exposure whereas persister cells cannot, we hypothesized previously that high nutrient levels during treatment would promote resistance evolution, while persistence would be favored at low nutrient levels [6]. Our evolution experiments indeed showed that resistance evolves at higher rates and attains higher levels when nutrient levels are high, a trend that was reproduced in simulated populations. The experimental data further suggest increased rates of persistence evolution in low-nutrient conditions, although this was not captured by the model. The fact that populations simulated under low nutrient concentrations and high antibiotic concentrations occasionally survive without evolutionary adaptation (Fig. 3; Fig. S4) suggests that the selective pressure in these conditions is underestimated by the model.

Provided that the nutrient concentration is sufficiently high, conditions with low antibiotic concentrations favor an evolutionary path leading to increased resistance over a path toward increased persistence, as resistant mutants have an advantage due to their ability to grow during treatment. Conversely, increased persistence seems to be favored over resistance in high antibiotic concentrations. Here, single-step resistant mutants are killed by the antibiotic, whereas persister cells tolerate a wider range of drug concentrations.

Many experimentally evolved populations showed an increase in both resistance and persistence, even at the clonal level. Although we cannot exclude the possibility that this is the result of two individual mutations, phenotypic measurements through time often show a simultaneous increase in both phenotypes, suggesting that pleiotropic mutations might be common.

For practical reasons, experimentally evolved populations (and hence also simulated populations) experienced a population bottleneck before treatment that is dependent on the nutrient level. Although this could potentially confound the observed nutrient effects, we verified *in silico* that this nutrient-dependent bottleneck does not significantly affect our conclusions (Fig. S5).

The bacterial populations under study are relatively small, as a consequence of the high throughput of our experimental evolution protocol as well as computational limitations. Our current model implementation aimed for a mutational supply matching the level in experimental populations, but this could have affected the resulting mutational patterns. Furthermore, larger populations are characterized by a larger mutational supply, implying that rare, large-effect mutations might arise more frequently. In such cases, a one-step, high-level resistance mutant might emerge and take over at high antibiotic doses, without requiring increased persistence for survival.

Even though our theoretical framework only consists of a set of relatively simple equations for growth and antibiotic killing, the emerging evolutionary patterns largely capture the predominant trends observed in experimentally evolved populations. Despite some discrepancies, this indicates that our basic model assumptions on growth and antibiotic survival of resistance and persistence mutants are sufficient to explain most of the observed evolutionary behavior. Nevertheless, certain limitations of our approach might need to be addressed in order to extrapolate model predictions to other settings. First, the model does not incorporate potential fitness costs of resistance and persistence mutations, which are usually observed experimentally as a growth defect in antibiotic-free conditions [13, 40]. Second, mutations that reduce resistance or persistence levels are not considered. This implies that some evolutionary paths which might be relevant in real populations, such as the reduction of persistence in favor of resistance [23], are not accessible to simulated populations. Third, the distribution of mutational effects is approximated using a set of discrete bins, only allowing for an incremental increase in resistance and persistence levels and assuming equal frequencies of resistance and persistence mutations. Modeling mutational effects with a continuous distribution would be more accurate, but computationally costly. Furthermore, accurate estimates of the frequency and effect sizes of resistance and persistence mutations, as well as pleiotropic mutations, require more in-depth genomic and phenotypic analyses.

In conclusion, we report a rich dataset and theoretical framework on resistance and persistence evolution in a broad range of antibiotic treatment conditions. Our results demonstrate that intermittent treatment schedules can select for both resistance and persistence, with the evolutionary route being highly dependent on the antibiotic dose and nutrient level during treatment. This high-level study can provide a basis for new, more detailed hypotheses regarding the mutational dynamics within evolving populations and the underlying molecular mechanisms, which could be tested using large-scale whole-genome sequencing. Finally, our experimental results and mathematical model not only provide fundamental insights into evolutionary dynamics under antibiotic stress but also might be relevant for the design of evolution experiments and antibiotic therapies.

## Supplementary Material

WindelsCooletal2024_SI_rev2_wrae070

## Data Availability

Raw data supporting this study are available on Zenodo (DOI: 10.5281/zenodo.7550302). The code generated for this study is available on GitHub (https://github.com/EtthelWindels/evo-model-PR).
