# Supplementary material for: Antibiotic dose and nutrient availability differentially drive the evolution of antibiotic resistance and persistence
Source: ISME J. 2024 May 1;18(1):wrae070. doi: 10.1093/ismejo/wrae070 (PMC11102087; doi:10.1093/ismejo/wrae070)
Supplement: WindelsCooletal2024_SI_rev2_wrae070 [file windelscooletal2024_si_rev2_wrae070.pdf]

Supplementary Information

Antibiotic dose and nutrient availability differentially drive the evolution of antibiotic resistance and persistence

Etthel M. Windels, Lloyd Cool, Eline Persy, Janne Swinnen, Paul Matthay, Bram Van den Bergh, Tom Wenseleers, Jan Michiels

Contents

Supplementary figures 2

Supplementary tables 7

Supplementary figures

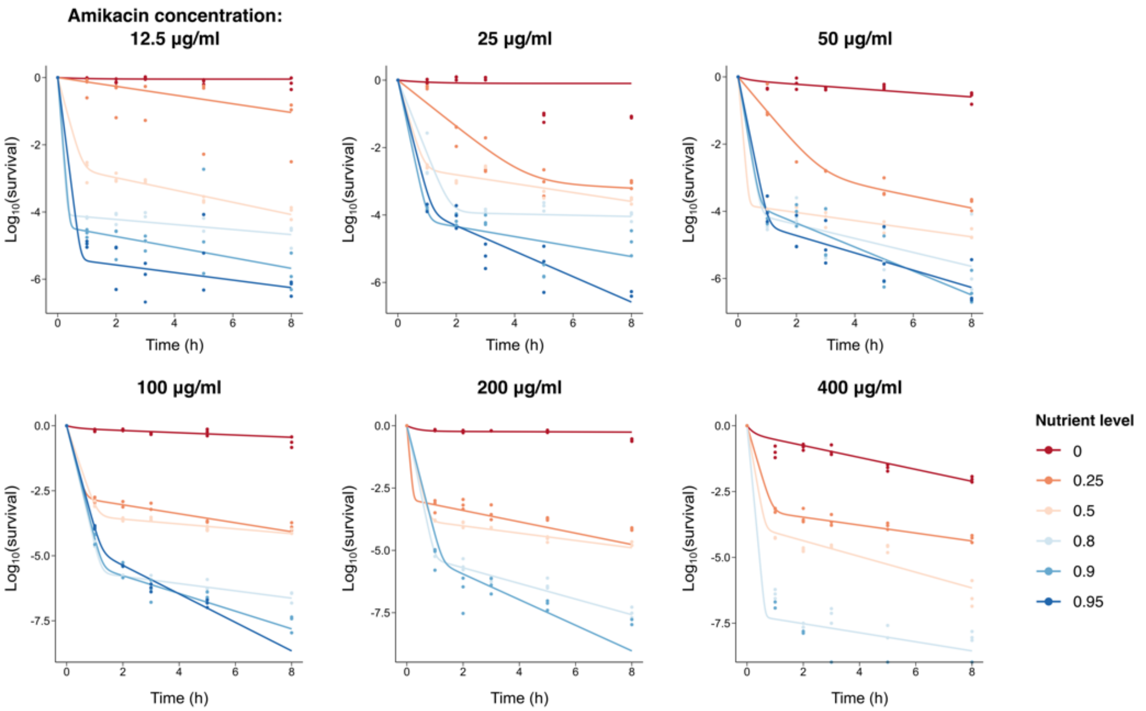

Fig. S1: Bacterial killing is promoted by increasing antibiotic and nutrient concentrations. Time-kill curves of the ancestral *E. coli* strain treated for 8 hours with varying amikacin concentrations and nutrient levels (n=3). Survival is depicted as the $\log_{10}$-transformed fraction of surviving cells. A biphasic exponential model was fitted onto the data for each condition (solid lines). The amikacin dose and nutrient level are both negatively correlated with survival after 8 hours (multivariable linear regression: P<0.01 for both main effects and interaction effect; Table S2). For higher antibiotic and nutrient concentrations, no survival was observed.

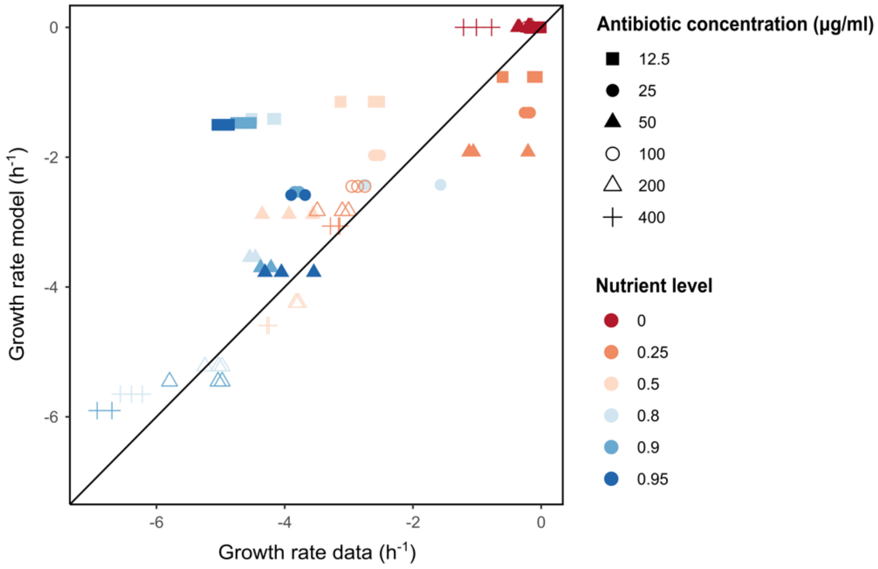

Fig. S2: Experimentally measured and modeled net growth rates are correlated. Experimentally measured net growth rates of the ancestral strain (Fig. S1) correspond well to the growth rates predicted by the model (see Materials and Methods) (Pearson correlation: r=0.83, P<0.001). Exceptions are the growth rates at 12.5 µg/ml, which are consistently lower in the model. All growth rates are expressed as changes in $\log_{10}$(cell density) per hour.

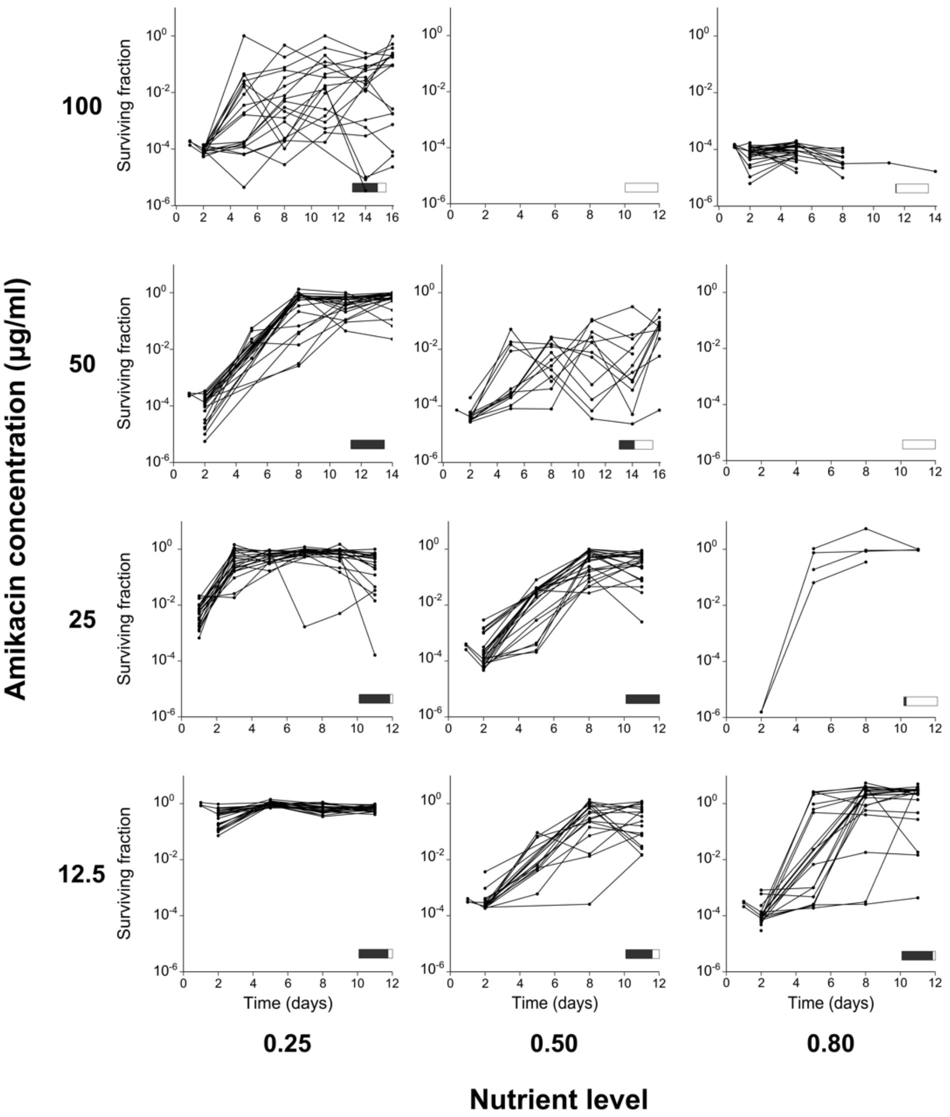

Fig. S3: Evolutionary dynamics of populations exposed to intermittent treatments with varying amikacin and nutrient concentrations. Fraction of cells surviving the daily, 5-hour treatment per population (n=24). Horizontal, filled bars depict the proportion of populations that did not go extinct during the evolution experiment. Empty graphs indicate that all populations went extinct. The amikacin concentration and nutrient level are positively correlated with the surviving fraction (multivariable linear regression: $P<0.001$ for both main effects; Table S3) as well as with the proportion of extinct populations (multivariable linear regression: $P<0.01$ for both main effects; Table S4).

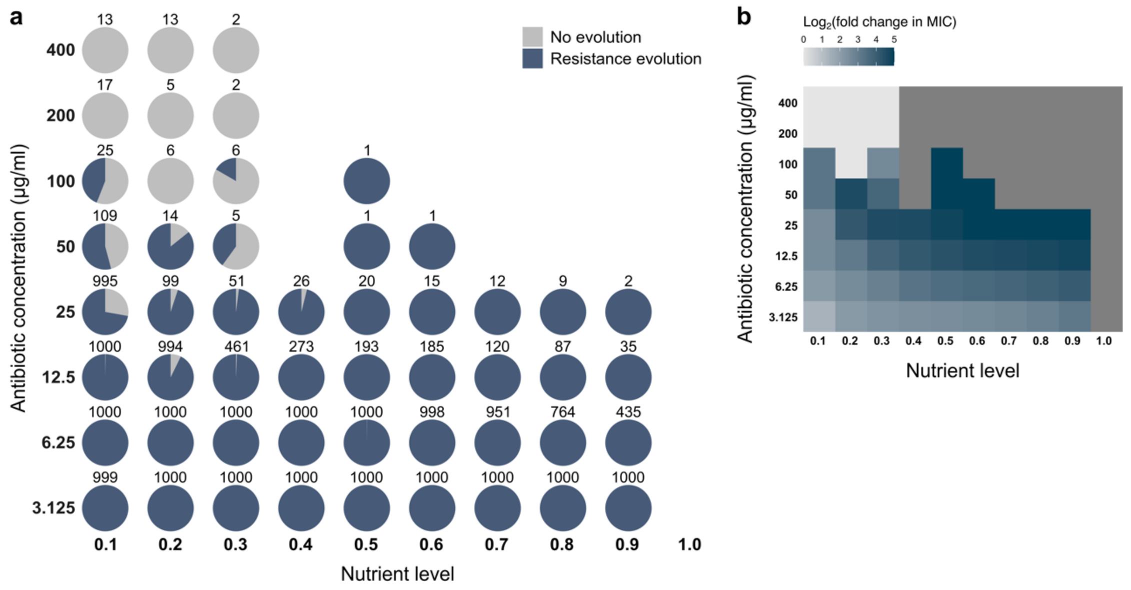

Fig. S4: Resistance evolution in simulated populations, in the absence of persistence evolution. a) Number of simulated populations evolving resistance in various treatment conditions. The persistence level was set equal to the ancestral level and was not affected by mutation. 1000 populations were simulated per condition. The total number of populations that did not go extinct is indicated. b) Resistance levels of evolved populations (expressed as $\log_2$-transformed fold changes relative to the ancestral level), averaged over all surviving populations per condition. Dark grey squares correspond to conditions where all populations went extinct. Note that at low nutrient levels, antibiotic killing is too slow to exert strong selection pressure, even at high antibiotic doses. This results in high survival despite relatively low resistance levels and could explain the lack of evolution in many of these populations.

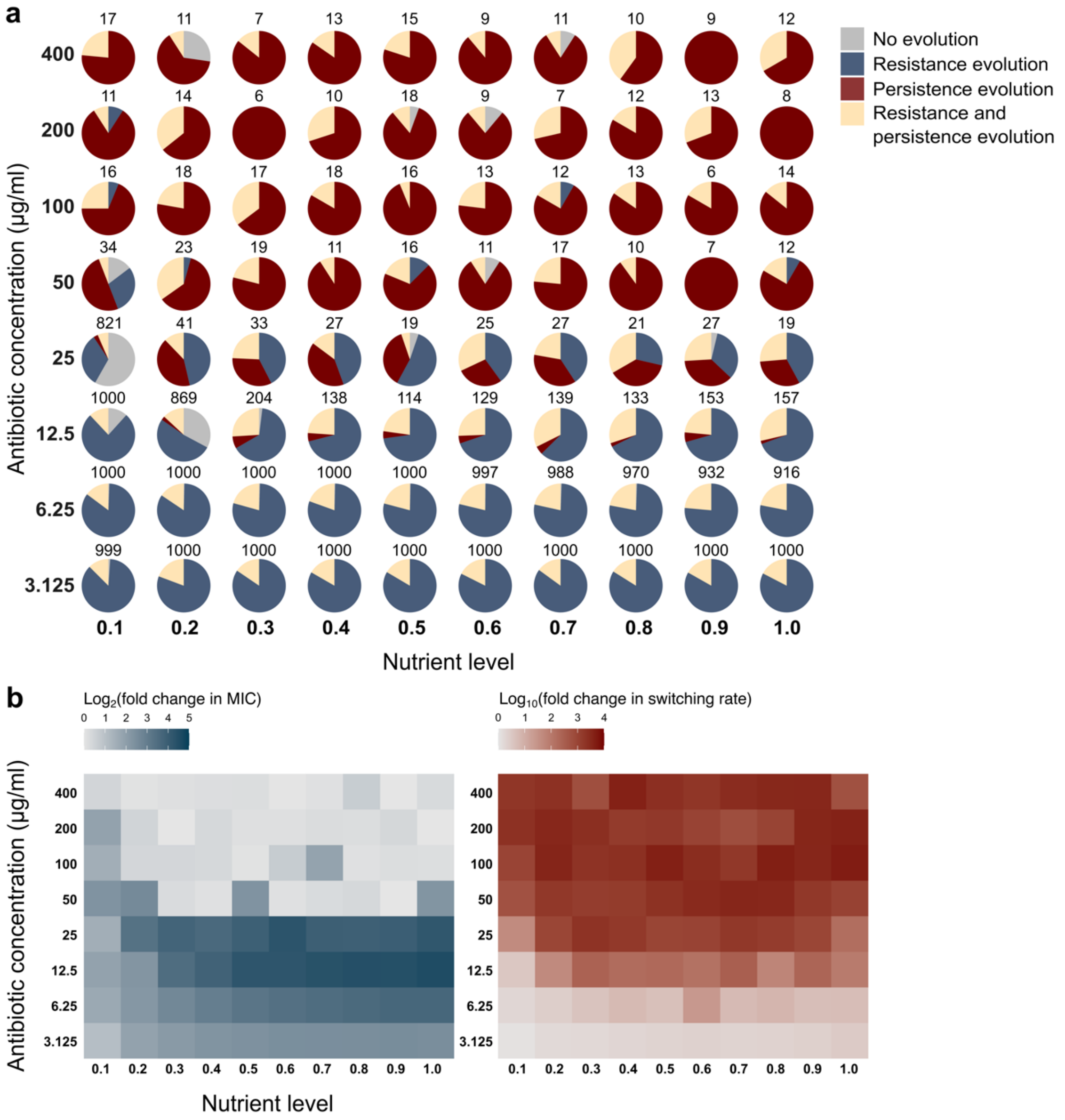

Fig. S5: Resistance and persistence evolution in simulated populations, assuming a nutrient-independent population bottleneck before treatment. Number of simulated populations evolving only resistance, only persistence, or both resistance and persistence, using a 1:2 dilution of all populations before treatment. 1000 populations were simulated per condition. The total number of populations that did not go extinct is indicated. b) Resistance and persistence levels of evolved populations (expressed as $\log_2$- resp. $\log_{10}$-transformed fold changes relative to the ancestral level), averaged over all surviving populations per condition. Except for the increased survival of populations at higher nutrient concentrations, overall trends are similar to simulations where the bottleneck before treatment is assumed to depend on the nutrient level (Fig. 3 of the main text).

Supplementary tables

Table S1: Overview of parameters used in the mathematical model of resistance and persistence evolution

Parameter	Description	Value	Reference
	Antibiotic treatment		
AB	Antibiotic concentration	3.125-400 μg/ml	
n	Relative nutrient level during treatment	0.1-1	
	Growth, killing, and persistence switching		
K	Carrying capacity	10^6 cells	
ψ_{max}	Maximum growth rate	0.6 h^{-1}	(13); this study
ψ_{min}	Minimum growth rate	-10 h^{-1}	this study
MIC_{anc}	MIC of ancestor	2 μg/ml	(13); this study
κ_{AB}	Hill coefficient antibiotic dose response curve	1	(27); this study
κ_n	Hill coefficient nutrient response curve	1	this study
$a_{anc,max}$	Maximum switching rate to persister state (ancestor)	$3.16*10^{-5}$ h^{-1} per cell	(9,13); this study
b_{max}	Maximum switching rate to regular state	0.1 h^{-1} per cell	(9,33); this study
	Resistance and persistence mutations		
m	Overall rate of resistance and persistence mutations	10^{-4} mutations per cell division	(13,21)

Table S2: Summary table for the linear regression model fitted onto the time-kill data (Fig. S1), with the dependent variable being the $\log_{10}$-transformed survival after 8 hours of treatment.

	**Estimate**	**Standard error**	***t* statistic**	***P* value**
Amikacin concentration	-2.94E-03	1.04E-03	-2.83	5.68E-03
Nutrient concentration	-5.27	2.97E-01	-17.71	< 2.00E-16
(Amikacin concentration) x (nutrient concentration)	-8.11E-03	1.93E-03	-4.20	6.32E-05

Table S3: Summary table for the linear regression model fitted onto the experimental evolution data (Fig. S3), with the dependent variable being the log_{10}-transformed fraction of cells surviving the 5-hour treatment in the evolved populations.

	**Estimate**	**Standard error**	***t* statistic**	***P* value**
Amikacin concentration	1.55E-02	3.67E-03	4.23	3.97E-05
Nutrient concentration	4.73	5.07E-01	9.32	< 2.00E-16

Table S4: Summary table for the linear regression model fitted onto the experimental evolution data (Fig. S3), with the dependent variable being the proportion of extinct populations.

	**Estimate**	**Standard error**	***t* statistic**	***P* value**
Amikacin concentration	8.11E-03	2.01E-03	4.04	1.41E-03
Nutrient concentration	1.36	3.56E-01	3.82	2.11E-03

Table S5: Summary table for the linear regression model fitted onto experimental evolution data (Fig. 1), with the dependent variable being log_2-transformed MIC of evolved populations.

	**Estimate**	**Standard error**	***t* statistic**	***P* value**
Amikacin concentration	2.74E-02	4.34E-03	6.32	4.30E-09
Nutrient concentration	3.65	6.02E-01	6.06	1.53E-08

Table S6: Summary table for the linear regression model fitted onto the experimental evolution data (Fig. 1), with the dependent variable being the log_{10}-transformed persistence level of evolved populations.

	**Estimate**	**Standard error**	***t* statistic**	***P* value**
Amikacin concentration	6.78E-04	4.15E-03	1.63E-01	8.70E-01
Nutrient concentration	-1.96	5.75E-01	-3.41	8.83E-04
